# Reproducibility and diagnostic performance of ADC for assessing response to neoadjuvant immunotherapy in muscle-invasive bladder cancer: a multi-ROI comparison analysis from the phase-Ib NABUCCO trial

**DOI:** 10.1186/s13244-026-02397-z

**Published:** 2026-10-13

**Authors:** Joyce Greidanus, Carlotta Bernabei, Kalina Chupetvloska, Georgios Agrotis, Laura S. Mertens, Bas W. G. van Rhijn, Henk G. van der Poel, Michiel S. van der Heijden, Regina G. H. Beets-Tan, Andrea Delli Pizzi, Thierry N. Boellaard, Stefano Trebeschi

**Affiliations:** 1 Department of Radiology, Netherlands Cancer Institute, Amsterdam, Netherlands; 2 Department of Surgical Oncology (Urology), Netherlands Cancer Institute, Amsterdam, Netherlands; 3 Department of Medical Oncology, Netherlands Cancer Institute, Amsterdam, Netherlands; 4 GROW School for Oncology and Reproduction, Maastricht University, Maastricht, Netherlands; 5 Department of Radiology, Santissima Annunziata Hospital, University “G. D’Annunzio”, Chieti, Italy; 6 Department of Urology, Amsterdam University Medical Centers, Amsterdam, Netherlands; 7 Netherlands Cancer Institute, Amsterdam, Netherlands; 8 Faculty of Health Sciences, University of Southern Denmark, Odense, Denmark; 9 ITAB—Institute for Advanced Biomedical Technologies, Gabriele d’Annunzio University of Chieti, Chieti, Italy

**Keywords:** Apparent diffusion coefficient, MRI biomarkers, Muscle-invasive bladder cancer, Neoadjuvant immunotherapy, Response assessment

## Abstract

**Objective:**

MRI is increasingly used to assess treatment response in muscle-invasive bladder cancer (MIBC), but only qualitatively. This study evaluates the reproducibility and predictive value of quantitative apparent diffusion coefficient (ADC), thought to be a non-invasive surrogate for tumor cell density, in patients with MIBC receiving neoadjuvant immunotherapy.

**Materials and methods:**

MRI data from 35 patients, enrolled in the Phase-Ib NABUCCO trial, were analyzed. ADC maps were derived from pre- and post-treatment MRI scans. Three independent radiologists assessed multiple regions of interest (ROIs) to evaluate inter-observer agreement and predictive performance for pathological complete response (pCR). ADC values were normalized using muscle tissue as a reference. Additionally, we analyzed the correlation between ADC and cell densities, derived from multiplex immunofluorescence (mIF) data.

**Results:**

ADC measurements from the whole-tumor ROI (*n* = 25) demonstrated the highest reproducibility (0.98, 95% CI = [0.95–0.99]), but lacked predictive value (AUC = 0.31, 95% CI = [0.12–0.53], *p* = 0.109). The most hypointense ROI (*n* = 22) showed the strongest predictive value for pCR (AUC = 0.80, 95% CI = [0.57–1], *p* = 0.025), though with limited reproducibility. The difference between baseline and follow-up ADC values in the hypointense region (*n* = 20) again showed the highest diagnostic accuracy (AUC = 0.80, 95% CI = [0.58–0.98], *p* = 0.015). Although muscle normalization is proposed to reduce scanner variability, it did not improve predictive performance in this cohort and occasionally reduced reproducibility. No significant correlation was found between whole-tumor ADC and cell density.

**Conclusion:**

Despite analyzing different measurement techniques for ADC values, none was simultaneously reproducible and predictive, nor provided insights into the biology of the tumor. While ADC shows promise in MIBC, there is an urgent need for more standardized, robust imaging biomarkers.

**Key Points:**

***Question*** Non-invasive biomarkers are urgently needed to guide bladder-preserving strategies in MIBC.

***Findings*** DWI-MRI shows promise, but standardized robust imaging biomarkers are essential for reliable response assessment.

***Critical relevance statement*** Standardized or AI-assisted ROI segmentation could improve ADC reproducibility and diagnostic accuracy in multicenter studies.

**Graphical Abstract:**

**Figure:**
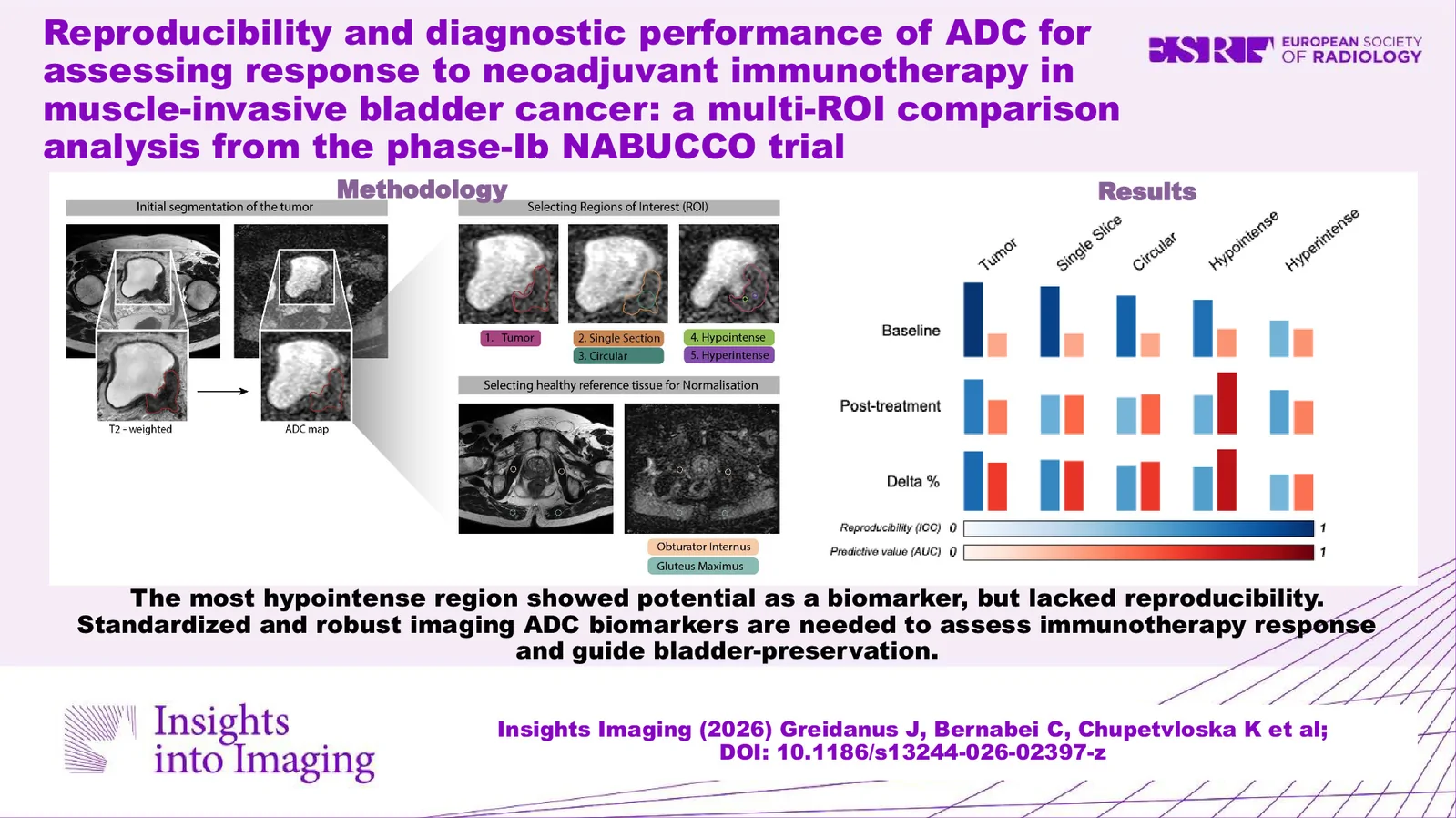

## Introduction

Standard treatment for patients with muscle-invasive bladder cancer (MIBC) consists of radical cystectomy (RC), which can be preceded by neoadjuvant therapy [1]. Organ-sparing treatment strategies after neoadjuvant treatment are under active investigation [2–5], driven by promising results from new systemic therapies, reporting high pathological complete response (pCR) rates of 42–46% [6–8]. To successfully deescalate locoregional treatment, the development of accurate, non-invasive methods to exclude residual tumor, is essential.

Magnetic resonance imaging (MRI) provides non-invasive assessment of tumor stage in bladder cancer through established staging systems like the American Joint Committee on Cancer (AJCC) Staging [9] and the Vesicle Imaging-Reporting and Data System (VI-RADS) [10]. However, beyond assessing muscle invasion, quantitative imaging biomarkers directly derived from the tumor have not yet been investigated in patients with MIBC undergoing neoadjuvant immunotherapy.

Multiparametric MRI (mpMRI), used in AJCC and VI-RADS, offers good soft-tissue contrast, but lacks the quantitative sensitivity needed to detect physiological changes associated with tumor response. Diffusion-Weighted Imaging (DWI) and its derivative, the Apparent Diffusion Coefficient (ADC), are used to quantify the motion of water molecules within tissues. Malignant lesions are associated with higher cellularity and high extracellular disorganization, causing restriction of water diffusion, resulting in a high signal intensity on DWI and, respectively, hypointensity on ADC [11–13].

ADC-derived parameters have demonstrated utility in staging, stratification, and treatment monitoring of immunotherapy across various cancers, including breast cancer [14], head and neck cancer [15], and melanoma [16]. For MIBC, preliminary studies suggest that quantitative ADC analysis may help assess response to neoadjuvant chemotherapy (NAC) [13, 17, 18]. However, ADC metrics have not yet been studied in the context of neoadjuvant immunotherapy. Unlike chemotherapy, which reduces tumor cellularity through direct cytotoxicity, immunotherapy acts via immune cell infiltration and inflammatory stromal remodeling, which may result in distinct diffusion properties and ADC response patterns [16]. Furthermore, prior studies have relied on single-reader approaches without systematic comparison of ROI delineation strategies, leaving inter-reader reproducibility and a standardized methodology for ROI placement unestablished. To support clinical implementation as a non-invasive imaging biomarker for treatment response assessment, ADC metrics must be robust, reproducible, and methodologically standardized. Therefore, this study addresses these gaps by evaluating the multi-reader reproducibility of competing ROI strategies and their diagnostic performance in predicting pCR in MIBC patients undergoing neoadjuvant immunotherapy, ultimately aiming to support organ-sparing management.

## Methods and materials

### Study population

**Fig. 1:**
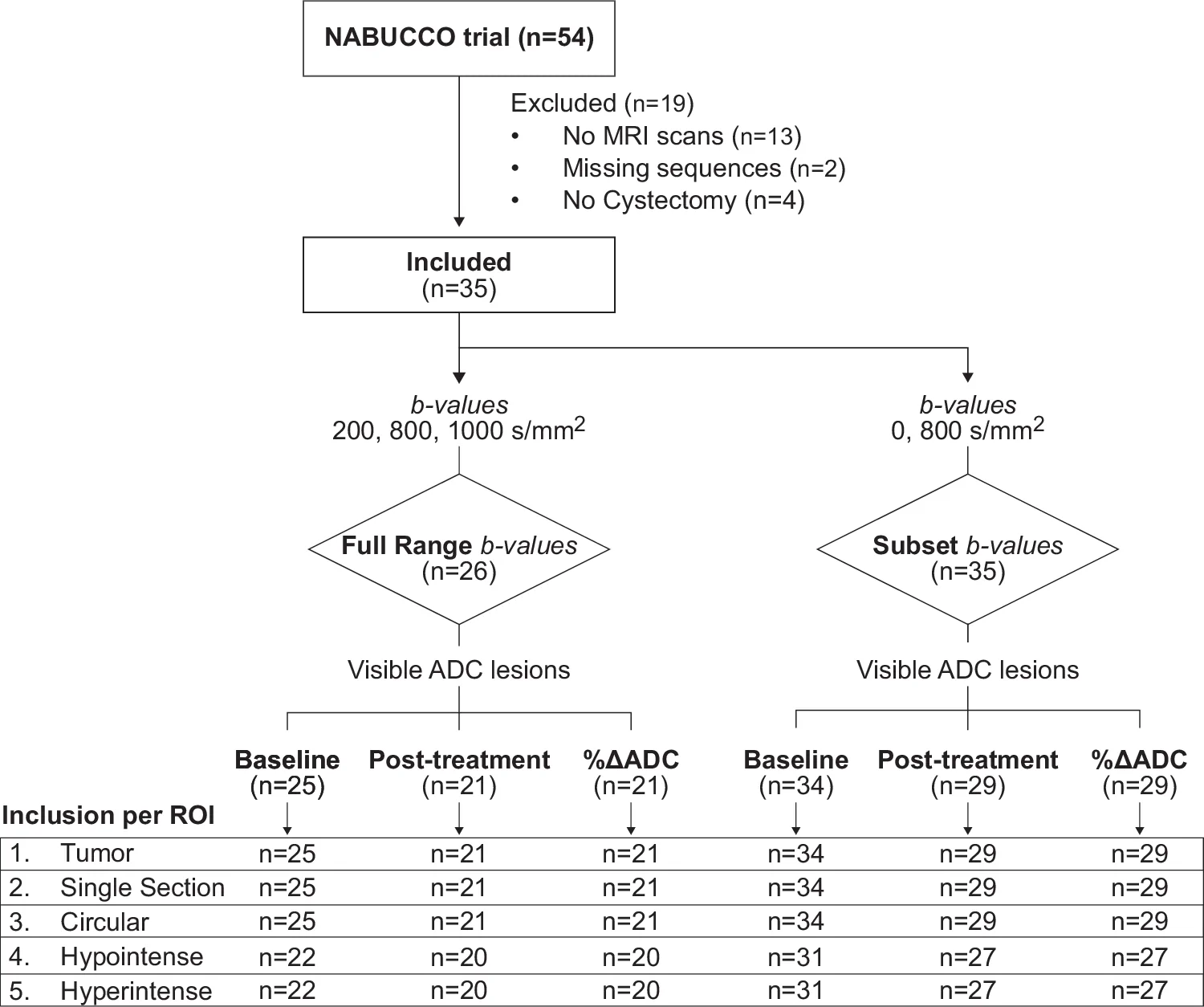
Flow diagram of patient inclusion and ROI placement inclusion

We retrospectively analyzed patients with histologically confirmed MIBC from the Phase-Ib NABUCCO trial (*n* = 54) [7, 8], which investigates the efficacy and safety of neoadjuvant immunotherapy (Ipilimumab + Nivolumab) followed by RC. Of these, 13 patients had no MRI scans available, and 2 had missing DWI sequences, leaving 39 patients with evaluable MRI data. An additional four patients were excluded because they did not undergo cystectomy, precluding pathological response assessment. Finally, 35 patients were included in the imaging analysis. Due to variation in DWI acquisition protocols across centers (see MRI Protocol below), two ADC datasets were generated and analyzed separately: full range *b*-values (*n* = 26) and Subset *b*-values (*n* = 35). ADC measurements were performed only in patients with a visible lesion on ADC maps, and the percentage change in ADC (%ΔADC) was calculated for patients with a visible lesion at both time points. The complete cohort flow and all denominators are summarized in Fig. 1.

### MRI protocol

Baseline MRI was performed after transurethral resection of bladder tumor (TURBT) and before immunotherapy, and post-treatment MRI after completion of immunotherapy and before RC. Scans were acquired using a 3.0 T Philips system (Achieva dStream or Ingenia). To ensure bladder distension, patients voided and subsequently drank 500 mL of water 30 min before scanning. T2-weighted images were obtained, the DWI protocol included four *b*-values (0, 200, 800, and 1000 s/mm²), and ADC maps were calculated based on the acquired *b*-values of 200, 800, and 1000 s/mm². Ten patients were scanned at another center, and DWI was acquired with only two *b*-values (0 and 800 s/mm²). As ADC values depend directly on the selected *b*-values and fitting procedure, maps generated from different *b*-value combinations are not directly comparable. To account for this variation, two ADC datasets were generated: full-range with *b* = 200–800–1000 s/mm^2^ (*n* = 26) and another using *b* = 0–800 s/mm^2^ (*n* = 35), both analyzed separately to assess protocol impact (Fig. 1 and Supplementary 1).

### Image processing and analyses

Three radiologists (C.B., K.C., and G.A.) independently reviewed scans using a custom 3D Slicer interface (v5.6.2). Prior to the study reads, all radiologists received a standardized instruction on the ROI placement methodology and completed one calibration case. Readers were blinded to the pathological outcomes and each other’s measurements. Cases were presented in randomized order; for each patient, baseline and post-treatment scans were reviewed consecutively to enable direct comparison, reflecting clinical practice.

**Fig. 2:**
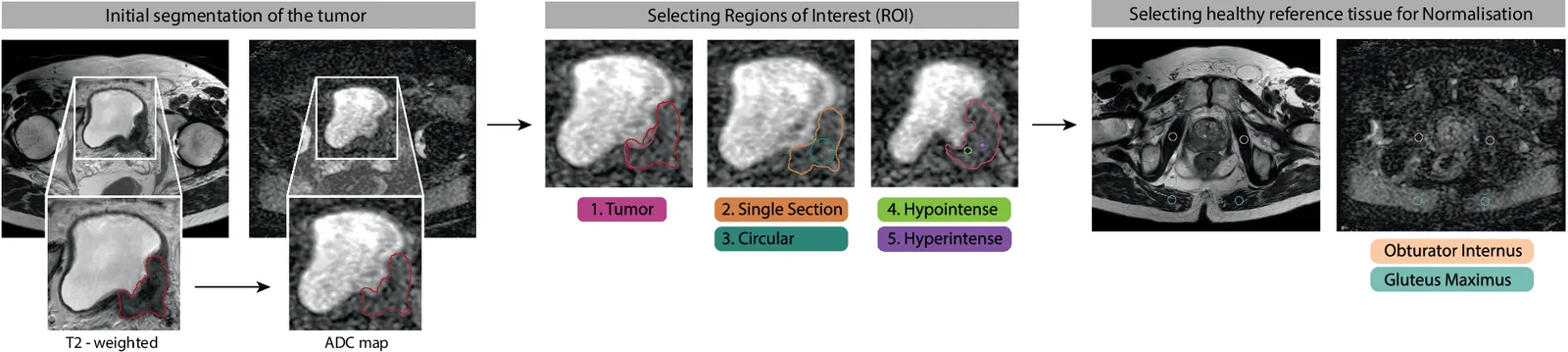
Schematic representation of the methodology for ADC analysis in MIBC. Initial tumor segmentation was performed on T2-weighted images and adjusted to the lesion signal on the ADC map (left). Multiple ROIs were selected on the ADC maps, including: (1) whole-tumor, (2) single-section, (3) circular, (4) most hypointense, and (5) most hyperintense ROIs (middle). For normalization of ADC values, reference ROIs were placed in healthy muscle tissue, specifically in the obturator internus and gluteus maximus (right)

The radiologists were presented with a T2-weighted image, ADC map, and the initial segmentation of the tumor (previously performed on the T2-weighted images but overlaid on top of the ADC map). The radiologists were tasked to perform the following ROI measurement on the ADC map, in order: (1) whole-tumor (adapting the segmentation to match the lesion signal on ADC), (2) largest cross-sectional lesion area, (3) maximum circular ROI within the lesion, (4) fixed 5 mm circular ROI on the region exhibiting the greatest restricted diffusion (visually the most hypointense area), and (5) fixed 5 mm circular ROI on visually the most hyperintense area (Fig. 2). Hypo- and hyperintense ROIs extending beyond the lesion boundaries were excluded. Radiologists segmented only visible lesions; in the absence of a detectable lesion, no ROI was defined, as reliable ADC assessment was not feasible. ADC values were extracted as median values per region of interest (ROI) drawn on ADC maps.

To quantify changes in ADC for response assessment, we computed the relative percentage change in median ADC value (%ΔADC) between baseline and post-treatment ADC values using the following formula:

$$\% \Delta {{ADC}}=\frac{{{ADC}}_{{post}}-{{ADC}}_{{baseline}}}{{{ADC}}_{{baseline}}}\times 100 \%$$

As an exploratory analysis, a normalization strategy was implemented to reduce variability in MRI intensities arising from different scanners, sequences, and parameters, using ADC values from healthy reference tissues [19]. Specifically, 10 mm circular ROIs were placed bilaterally within the gluteus maximus muscles and the obturator internus muscles (see Fig. 2). ROIs were carefully positioned to avoid artifacts, blood vessels, fatty muscle tissue, and atrophic muscle regions whenever feasible. Normalized ADC (nADC) was calculated as:

$${nADC}=\frac{{Median}\,{ADClesion}}{{Average}\,{ADCmuscle}\,}$$

### Pathological reference standard

Treatment response was defined by pathological T-stage (ypT) post-cystectomy. Patients with ypT0 or ypTcis were considered pCR, while those with residual tumor (≥ ypTa) were considered non-pCR.

As an exploratory analysis, ADC values were also compared with tumor and immune cell densities derived from multiplex immunofluorescence (mIF) data, obtained from TURBT and resected bladder specimens, given that ADC is considered a non-invasive surrogate for tumor cell density [12]. The mIF is used to characterize the tumor microenvironment (TME) at single-cell resolution, and provides cell count and density estimations of immune cells (CD8+, CD103+, and TCF1+) and tumor cells (PanCK+).

### Statistical analysis

The primary analyses comprised inter-observer agreement and predictive performance. Inter-observer agreement was assessed using intraclass correlation coefficients (ICC[2,1]) (average-rater reliability (ICC[2,k]) in Supplementary 2). To minimize reader variability, consensus ADC values were calculated as the mean of the three radiologists’ median values. The area under the receiver operating characteristic curve (ROC-AUC) and Mann–Whitney *U*-tests were used to evaluate the predictive performance of baseline, post-treatment, and %ΔADC for pCR (ypT0/ypTcis). As exploratory analyses, the agreement and predictive performance of nADC was evaluated using the same approach, and Spearman’s rank correlation assessed associations between ADC and mIF-derived cell densities (total cell density, tumor cell density, and immune cell density).

## Results

### Patient characteristics

Of 54 enrolled NABUCCO trial patients, 35 were eligible for imaging analysis after exclusion of 19 patients (no MRI scans, *n* = 13; missing DWI sequences, *n* = 2; no cystectomy, *n* = 4). Full-range ADC analysis was possible in 26 patients; all 35 were evaluable using subset *b*-values. Patient characteristics of both groups are presented in Table 1. Among the 26 full-range patients, 14 (53.8%) achieved pCR, and 12 (46.2%) were classified as non-pCR. After excluding patients without visible lesions at baseline (*n* = 1) or post-treatment (*n* = 5), 25 patients were evaluable at baseline, 21 post-treatment, and 21 for %ΔADC (Fig. 1).

**Table 1:** Patient and tumor characteristics

Patient characteristics	NABUCCO	
	Full range *b* = 200–800–1000 s/mm^2^ (*n* = 26)	Subset *b* = 0–800 s/mm^2^ (*n* = 35)
Median age, years [IQR]	71 [57–81]	69 [57–81]
Gender, *n* (%)		
Male	19 (73%)	24 (69%)
Female	7 (27%)	11 (31%)
Clinical T stage, *n* (%)		
cT2	0	2 (6%)
cT3	15 (58%)	19 (54%)
cT4	11 (42%)	14 (40%)
Time interval, days [IQR]		
TURBT to baseline scan	54 [43–62]	50 [36–54]
Baseline scan to immunotherapy	6 [1–8]	9 [2–11]
Immunotherapy to post-treatment scan	19 [14–22]	18 [13–21]
Post-treatment scan to RC	16 [10–21]	16 [10–21]
Pathological T stage, *n* (%)		
pT0	12 (46%)	15 (43%)
pTcis	2 (8%)	4 (11%)
pTa	1 (4%)	1 (3%)
pT1	0	2 (6%)
pT2	0	1 (3%)
pT3	6 (23%)	6 (17%)
pT4a	5 (19%)	6 (17%)

*IQR* interquartile range, *TURBT* transurethral resection of bladder tumor, *RC* radical cystectomy

The median volume of the whole-tumor ADC ROI at baseline was 17.16 mL (range 0.36–19.47), with no significant difference between patients with pCR (8.89 mL, IQR = [1.23–33.17]) and non-pCR (23.98 mL, IQR = [11.22–32.55], *p* = 0.27). After neoadjuvant therapy, a significant difference was observed between pCR (1.12 mL, IQR = [0.08–4.67]) and non-pCR (18.26 mL, IQR = [9.78–31.84], *p* = 0.002).

### Baseline ADC is not predictive

**Fig. 3:**
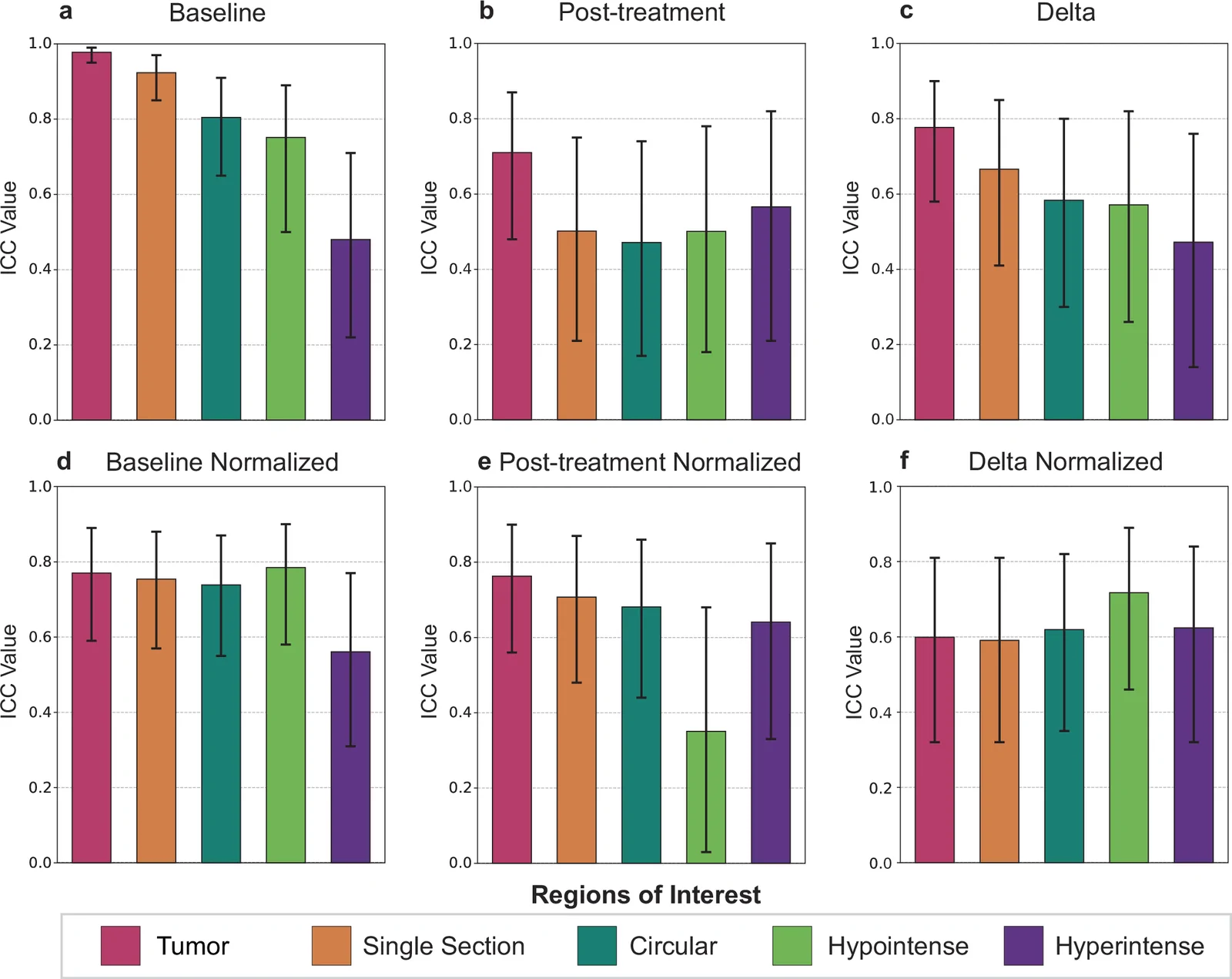
Inter-observer variability of ADC measurements across different ROI strategies and timepoints. Bar plots show ICCs (ICC[2,1]) with 95% confidence intervals (CIs) for five ROI methods at (**a**) baseline (*n* = 25), (**b**) post-treatment (*n* = 21), and (**c**) as the percentage change (%ΔADC) (*n* = 21). **d**–**f** Display the corresponding ICCs for nADC values using muscle tissue as reference

**Fig. 4:**
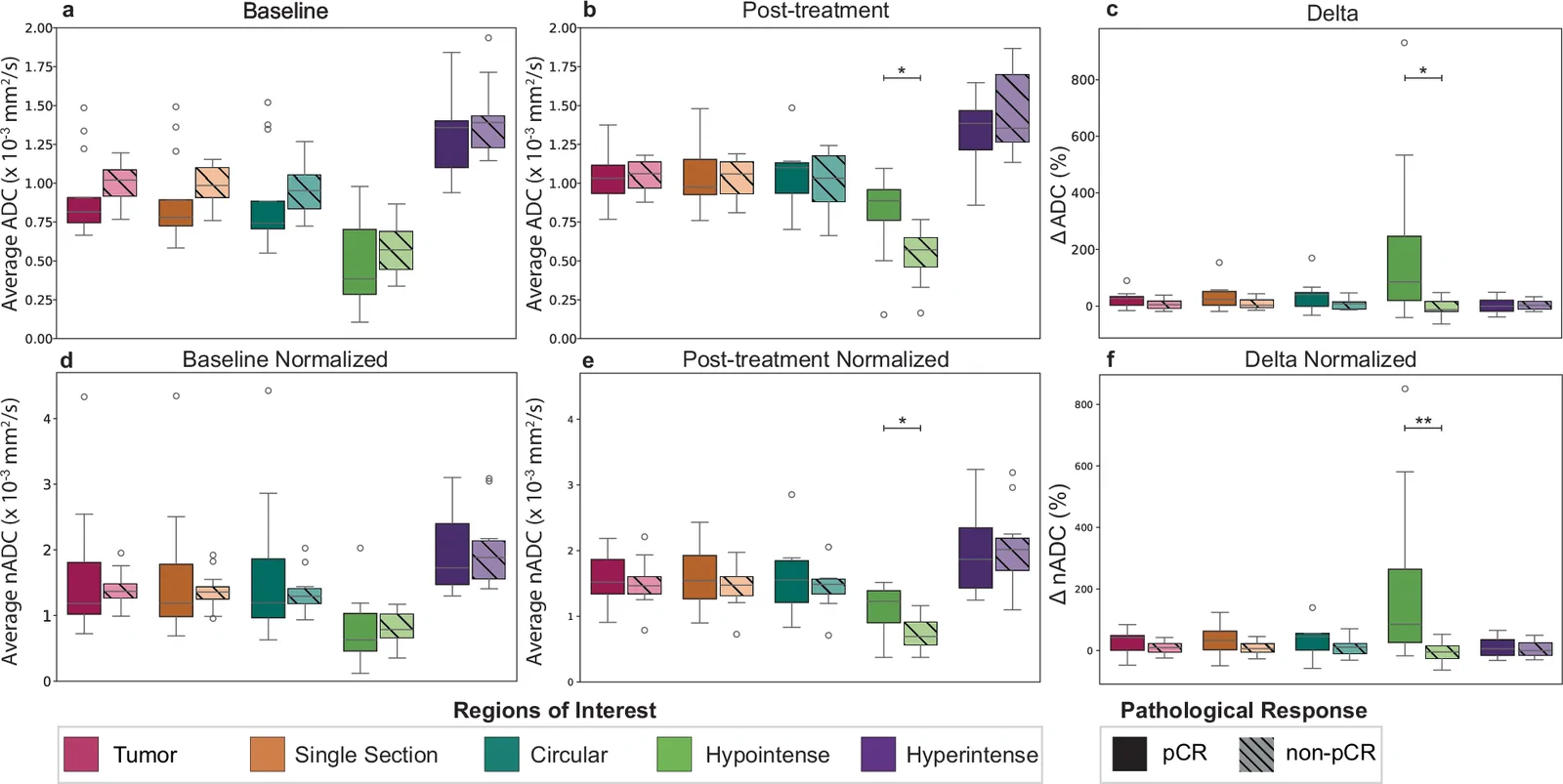
Boxplots of ADC values across ROIs, timepoints, and pathological response. Consensus median ADC values (mean of three readers) are shown for five ROI types at (**a**) baseline (*n* = 25), (**b**) post-treatment (*n* = 21), and (**c**) as the percentage change (%ΔADC) (*n* = 21). **d**–**f** Show the same metrics after normalization to muscle tissue (nADC). Boxplots represent the interquartile range (IQR), horizontal lines indicate the median, and whiskers extend to 1.5 × IQR. pCR was defined as ypT0/ypTcis; non-pCR as ≥ ypTa. *p*-values were calculated using the Mann–Whitney *U*-test; ^*^p < 0.05; ^**^p < 0.01, ^***^p < 0.001

**Fig. 5:**
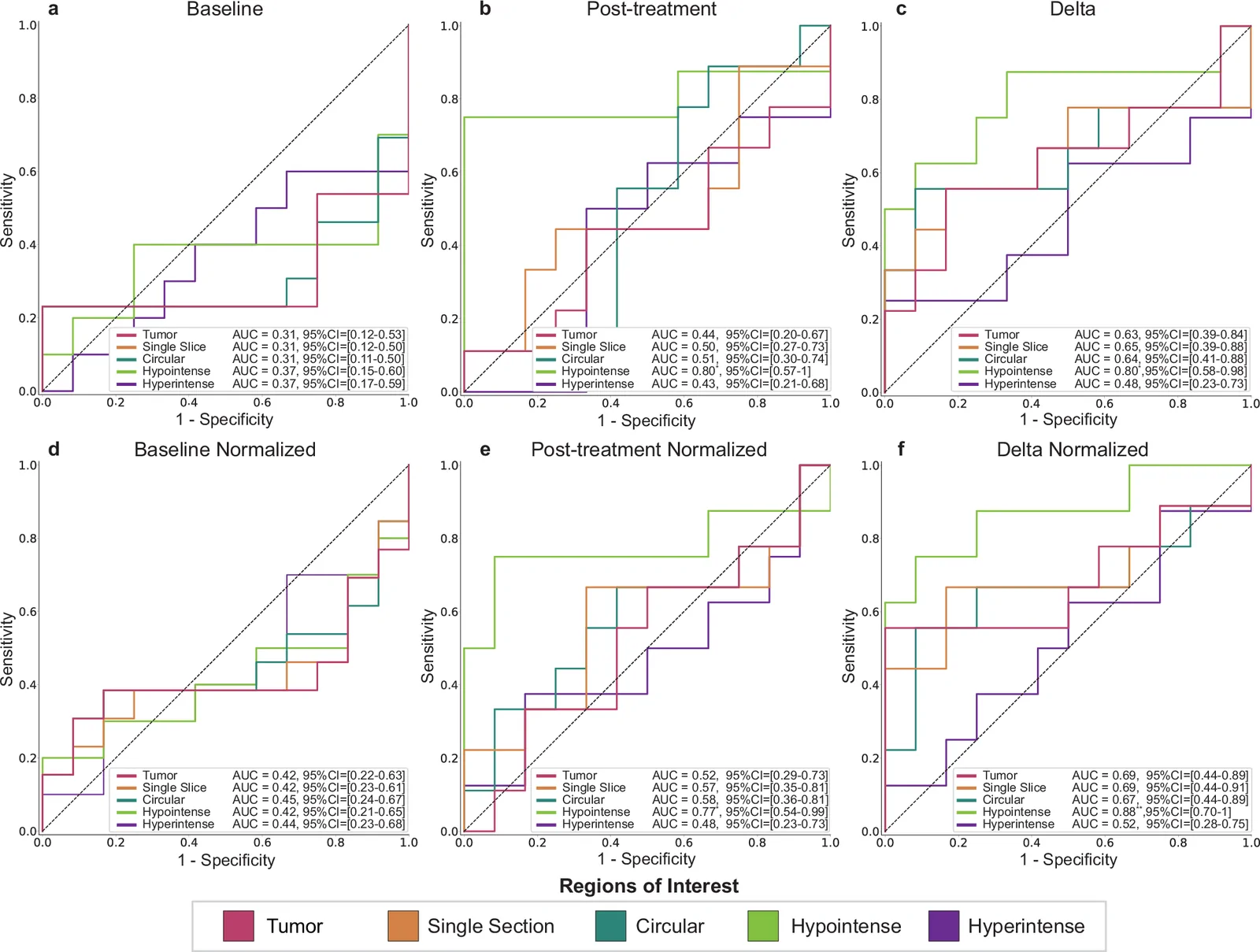
ROC curves for the prediction of pCR using ADC metrics across ROI strategies and timepoints. ROC curves with corresponding area under the curve (AUC) and 95% CIs are shown for five ROI types at (**a**) baseline (*n* = 25), (**b**) post-treatment (*n* = 21), and (**c**) percentage change (%ΔADC) (*n* = 21). **d**–**f** Present the same analysis after normalization (nADC). pCR was defined as ypT0/ypTcis; non-pCR as ≥ ypTa. Significance test ^*^p < 0.05; ^**^p < 0.01, ^***^p < 0.001

Among patients with a visible lesion at baseline (*n* = 25), the highest ICC score was reached by whole-tumor measurements (ICC = 0.98, 95% CI = [0.95–0.99]), followed by single-section (ICC = 0.92, 95% CI = [0.85–0.97]), circular (ICC = 0.80, 95% CI = [0.65–0.91]), hypointense (*n* = 22, ICC = 0.75, 95% CI = [0.50–0.89]), and the hyperintense ROI (*n* = 22, ICC = 0.48, 95% CI = [0.22–0.71]) (see Fig. 3a). Baseline ADC values were non-significantly lower in pCR patients across all ROIs, with modest predictive performance (best AUC = 0.37*, 95% CI = [0.15–0.60], *AUC = 0.63 inverted, *p* = 0.31) (Table 2, and Figs. 4a and 5a).

**Table 2:** Median ADC values for baseline, post-treatment and the percentage change over time (%ΔADC)

ROI	Baseline			Post-treatment			%ΔADC		
	pCR *n* = 13	Non-pCR *n* = 12	*p*-value	pCR *n *= 9	Non-pCR *n* = 12	*p*-value	pCR *n* = 9	Non-pCR *n* = 12	*p*-value
Median ADC									
Tumor	0.82 [0.75–0.91]	1.02 [0.91–1.09]	0.12	1.03 [0.93–1.12]	1.06 [0.97–1.14]	0.64	27.10 [2.99–33.80]	5.19 [−7.50 to 18.84]	0.34
Single section	0.78 [0.73–0.89]	0.99 [0.91–1.10]	0.11	0.98 [0.93–1.15]	1.06 [0.93–1.14]	1.00	23.57 [2.89–51.42]	3.11 [−5.31 to 22.06]	0.27
Circular	0.74 [0.71–0.88]	0.95 [0.84–1.05]	0.11	1.10 [0.94–1.13]	1.03 [0.88–1.17]	0.97	40.17 [−0.61–48.05]	8.44 [−9.25 to 15.37]	0.30
Hypointense^*^	0.38 [0.29–0.70]	0.57 [0.45–0.69]	0.31	**0.89 [0.76–0.96]**	**0.57 [0.46–0.65]**	**0.025**	**84.81 [19.67–246.59]**	**−12.55 [−17.80 to 16.47]**	**0.025**
Hyperintense^*^	1.36 [1.10–1.40]	1.39 [1.23–1.43]	0.31	1.39 [1.21–1.47]	1.35 [1.26–1.70]	0.62	−0.39 [−18.14 to 20.63]	2.60 [−9.96 to 16.99]	0.91
Median nADC									
Tumor	1.18 [1.02–1.81]	1.37 [1.27–1.49]	0.53	1.52 [1.34–1.86]	1.46 [1.35–1.61]	0.91	43.22 [0.776–48.69]	9.60 [−5.51 to 23.73]	0.17
Single section	1.19 [0.98–1.78]	1.36 [1.25–1.44]	0.53	1.54 [1.26–1.93]	1.47 [1.32–1.61]	0.59	32.71 [2.45–62.73]	6.23 [−4.37 to 22.28]	0.15
Circular	1.19 [0.97–1.86]	1.29 [1.18–1.41]	0.68	1.56 [1.21–1.85]	1.49 [1.34–1.57]	0.55	45.30 [1.50–56.18]	10.88 [−10.06 to 23.11]	0.21
Hypointense^*^	0.62 [0.46–1.03]	0.79 [0.66–1.02]	0.58	**1.23 [0.90–1.39]**	**0.69 [0.56–0.92]**	**0.045**	**84.99 [26.36–264.01]**	**−4.82 [−25.20 to 15.18]**	**0.004**
Hyperintense^*^	1.73 [1.47–2.40]	1.88 [1.56–2.14]	0.67	1.87 [1.43–2.35]	2.02 [1.70–2.19]	0.91	5.24 [−15.79 to 35.11]	−0.19 [−15.49 to 25.22]	0.91

For both the raw ADC and the nADC values. Mean ADC values are given in ×10^−3^ mm^2^/s with 95% CIs. *p*-values refer to comparison between pCR and non-pCR, tested with the Mann–Whitney *U*-test. Significant results (*p* < 0.05) are highlighted in bold

^*^ The hypointense and hyperintense have a different patient count for pCR: Baseline *n* = 10, post-treatment *n* = 8, %ΔADC *n* = 8

Using the same baseline cohort (*n* = 25; *n* = 22), normalization did not improve inter-observer agreement nor predictive performance (Table 2, and Figs. 3d, 4d, and 5d). Obturator internus muscle normalization yielded higher reproducibility over gluteus muscles (obturator ICC = 0.63–0.70 vs gluteus ICC = 0.23–0.29), with strong bilateral correlation (Pearson’s *r* = 0.796, *p* < 0.001) vs gluteus muscles (Pearson’s *r* = 0.359, *p* = 0.078). Normalization was therefore performed using the mean bilateral obturator internus ADC.

### Post-treatment hypointense ROI is predictive but not reproducible

Among patients with a visible lesion at post-treatment (*n* = 21), inter-observer agreement was lower compared to baseline. Whole-tumor ROI again reached the highest reproducibility across readers (ICC = 0.71, 95% CI = [0.48–0.87]), while all other ROIs scored below 0.60 (Fig. 3b).

The highest predictive performance was observed by the hypointense ROI (*n* = 20, pCR ADC = 0.89 × 10^−3^ mm^2^/s vs non-pCR = 0.57 × 10^−3^ mm^2^/s, *p* = 0.025; AUC = 0.80, 95% CI = [0.57–1]). All other ROIs yielded non-significant differences with AUCs between 0.43–0.51 (Table 2; Figs. 4b and 5b).

Unlike at baseline, normalization improved overall inter-observer agreement, except for the hypointense ROI (*n* = 20, ICC = 0.35, 95% CI = [0.03–0.68] vs ICC = 0.78, 95% CI = [0.58–0.90] at baseline (Fig. 3e)). Similarly to baseline, obturator internus normalization reached higher inter-observer agreement compared to gluteus muscle (obturator ICC 0.76–0.85 vs gluteus ICC 0.45-0.63), with strong bilateral correlation Pearson’s *r* = 0.729, *p* < 0.001). (The normalized hypointense ROI remained the only significant predictor (pCR ADC = 1.23×10^−3^ mm^2^/s vs non-pCR = 0.69 × 10^−3^ mm^2^/s, *p* = 0.045; AUC = 0.77, 95% CI = [0.54–0.99]) (Table 2; and Figs. 4e and 5e).

### Potential use for longitudinal measurements

Among patients with a visible lesion at both timepoints (*n* = 21), %ΔADC showed a drop in inter-reader agreement similar to the post-treatment values. Whole-tumor ADC remained the highest inter-reader agreement (see Fig. 3c). Also, similar to the post-treatment median ADC values, the hypointense ROI showed a significant difference between pCR and non-pCR patients (*n* = 20, respectively, +84.81% vs −12.55%, *p* = 0.025; AUC = 0.80, 95% CI = [0.58–0.98]). The other ROIs ranged between 0.48 and 0.65 AUC, with no significant difference between pCR and non-pCR (Table 2; and Figs. 4c and 5c).

Similar trends were observed after normalization, where the most hypointense ROI scored the highest predictive value (AUC = 0.88, 95% CI = [0.70-1], *p* = 0.004) (see Figs. 4f and 5f) and the inter-reader agreement increased (ICC = 0.72, 95% CI = [0.46–0.89]) (Fig. 3f).

### Hypointense ROI not reflected by 10th percentile

The hypointense ROI shows the highest predictive power, but it has low agreement between radiologists. Whole-tumor ROI shows the highest reproducibility amongst radiologists, but not the highest predictive power. Therefore, we performed an exploratory analysis to evaluate whether the 10th percentile of the ADC values extracted from the whole-tumor ROI could be a more objective surrogate of the hypointense ROI. Significant correlations were observed between hypointense ROI values and both the post-treatment 10th percentile measurements (Spearman’s *r* = 0.58, *p* = 0.007) and the delta over time (Spearman’s *r* = 0.78, *p* < 0.001). The 10th percentile reached an ICC of 0.65 (95% CI = [0.4–0.83]) and the AUC was 0.45 (95% CI = [0.20–0.70]) for post-treatment, while for delta values, the ICC = 0.36 (95% CI = [0.09–0.65]) and AUC = 0.64 (95% CI = [0.36–0.88]).

### No impact when using fewer *b*-values

These analyses were performed on *n* = 35 patients (Supplementary 3). For this analysis, we used the raw and nADC values, based on the obturator internus muscle. Most results aligned with the results obtained using ADC maps with b200–800–1000. Whole-tumor ADC and nADC showed the highest inter-observer agreement at both baseline and post-treatment, and the hypointense ROI showed significant predictive value at post-treatment and for the difference over time. The only difference we observed was for the circular ROI of the %ΔnADC values, which showed a significant difference between pCR and non-pCR (33.48% vs 3.48%, *p* = 0.019; AUC = 0.76, 95% CI = [0.59–0.91]).

### Weak correlation to cell density

**Fig. 6:**
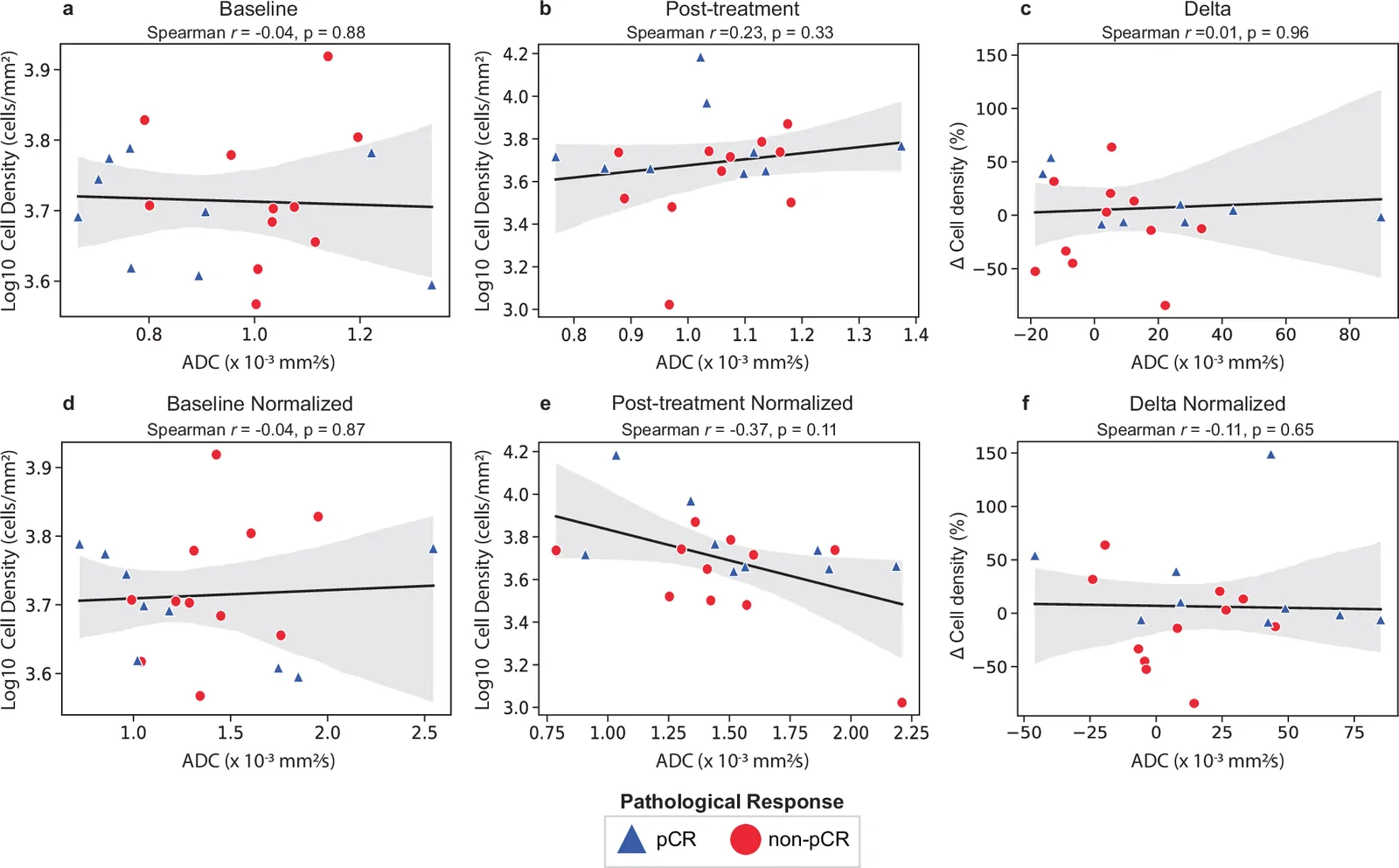
Exploratory correlation between whole-tumor ADC metrics and total cell density derived from mIF (*n* = 20). Scatterplots display the relationship between whole-tumor ADC values (×10⁻³ mm²/s) and total cell density (cells/mm²) at (**a**) baseline, (**b**) post-treatment, and (**c**) percentage change (%ΔADC). **d**–**f** Show the corresponding correlations for nADC. Data points are colored by pathological response: pCR (ypT0/ypTcis, blue) and non-pCR (≥ ypTa, red). Correlation coefficients (*r*) and *p*-values were calculated using Spearman’s rank correlation

Among the 20 patients with both ADC measurements and mIF data available, no significant correlations were observed for tumor cell density or total cell density (see Fig. 6). Only differences over time showed significant correlation between whole-tumor ADC and the cell density of the negative cells not covered by the panel (CD8-, CD103-, TCF1-, PanCK-, Spearman’s *r* = 0.56, *p* = 0.025) (Supplementary 4).

## Discussion

In this study, we evaluated the reproducibility and predictive value of quantitative ADC values in patients with MIBC treated with neoadjuvant immunotherapy. Whole-tumor ROI demonstrated the highest inter-observer agreement, making it the most reproducible ROI for ADC extraction, but its predictive value for response was limited. In contrast, the hypointense ROI demonstrated better predictive value post-treatment but lacked reproducibility. Baseline ADC values were consistently lower in pCR patients, though they had little to no discriminative power. Normalization did not improve the predictive value and was sometimes detrimental to reproducibility.

The whole-tumor ROI demonstrated the highest reproducibility, likely due to its larger size and comprehensive coverage, which reduces the impact of small positional variations. In contrast, the hypointense ROI, despite lower reproducibility, showed high predictive performance. This may be attributed to its focus on regions with the highest diffusion restriction, which corresponds to areas of high cellularity and potentially viable tumor cells. Conversely, whole-tumor measurements may include more heterogeneous tissue components, such as edema, fibrosis, or immune cell infiltration, that can dilute the tumor-specific signal and reduce diagnostic specificity. This highlights a fundamental trade-off in quantitative imaging biomarker development: more biologically sensitive measurements may inherently be less reproducible than volumetric approaches that gain stability at the cost of diagnostic specificity [20].

At baseline, patients achieving pCR exhibit lower ADC values compared to non-pCR. Similar patterns were observed by Yoshida et al for chemoradiation sensitivity in MIBC [21], consistent with what has been observed in other cancer types [22, 23]. However, opposite trends have also been observed [13], and discriminative power in our cohort was limited. Notably, baseline MRI was acquired after TURBT, which may introduce postoperative inflammation and edema that confound ADC measurements [18, 24, 25]. Although our cohort met the recommended minimum two-week interval between TURBT and MRI [10] (mean 51.6 days), residual post-surgical tissue changes cannot be fully excluded.

In accordance with previous research by Necchi et al [26], the whole-tumor post-treatment results show no difference between pCR and non-pCR. When looking at the change in ADC value after treatment (%ΔADC), similar patterns of increased ADC values over time were found in patients with MIBC achieving pCR after NAC [13, 17, 18]. An increase in ADC values after treatment has been attributed in literature to potential mechanisms, such as therapy-induced cellular damage and compromised cell membrane integrity. These effects may reduce tumor cell density and increase extracellular space, thereby decreasing diffusion restriction [13, 23]. However, following immunotherapy, the TME often exhibits stromal remodeling and immune cell infiltration around the residual disease, which could also influence ADC measurements and potentially affect the ability to accurately reflect residual tumor characteristics [27].

As an exploratory analysis, we examined the correlation between ADC measurements and cell densities (total, tumor, and immune) derived from mIF data. No significant correlations were found with tumor cell density or total cell density. This likely reflects spatial and temporal mismatch between the two modalities: histopathological analysis assesses a small tissue section at a single timepoint, whereas whole-tumor ADC measurements capture heterogeneous regions. Additionally, immune cell infiltration following immunotherapy exhibits complex spatial and temporal heterogeneity. Therefore, imaging and tissue sampling performed at different timepoints in the immune response may capture different stages of infiltration, which can weaken the correlation between the measurements [28].

ADC measurements are influenced by scanner-related and patient-specific variability, which normalization may help mitigate [29, 30]. In this study, we compared two muscle ROIs for normalization, the obturator internus and the gluteus maximus, with the obturator internus demonstrating higher agreement. This may be due to the obturator internus being a smaller muscle, allowing less variation in ROI placement compared to the larger gluteus muscle. Normalization did not significantly improve overall agreement, nor did it enhance discriminative power or inter-center reproducibility. These results underscore the need for standardized normalization protocols before implementing ADC normalization in bladder cancer assessment. Although the hypointense ROI lacked reproducibility, its significant predictive value suggests potential utility in future clinical and research settings. Our exploratory analysis showed that the 10th percentile of the whole-tumor ROI, as a more objective surrogate, correlated with the hypointense ROI but did not replicate its predictive performance, suggesting that the radiologists’ visual selection captures spatial information not fully reflected by percentile-based summary statistics. Standardized, AI-assisted segmentation techniques could enhance reproducibility and support the use of targeted ROIs in multicenter studies, addressing current challenges in ADC measurement standardization and potentially improving diagnostic accuracy [31].

This study had several limitations. First, this study was performed on a small dataset, which impacts the statistical power and the ability to detect true effects. Although we included a multicenter-based subanalysis, a larger, multicenter study is necessary to confirm our findings and improve generalizability. Second, baseline MRI was acquired after TURBT, which is a confounder for baseline ADC measurements, as explained before. Third, treatment response was solely based on pathological T-stage after cystectomy. We did not focus on the condition of the lymph nodes, because the added value of MRI for accurately assessing lymph nodes remains suboptimal [32]. Fourth, not all patients could be evaluated due to the absence of a detectable DWI signal, limiting the feasibility of quantitative ADC measurements. At baseline, this was attributed to the effects of radical TURBT, while post-treatment, a complete response could lead to a fully normalized MRI scan. With no detectable DWI or ADC signal, radiologists were unable to define an ROI for analysis. Despite these limitations, tumor volume differed significantly between patients with pCR and non-pCR, highlighting the potential of combining volume measurements with other imaging biomarkers [33]. Importantly, no single ADC strategy achieved sufficient combined reproducibility and predictive performance to support treatment de-escalation. These findings should be considered hypothesis-generating, identifying the hypointense ROI as promising but not yet clinically actionable. AI-assisted segmentation could help standardize targeted ROI placement, and the combination of ADC with complementary biomarkers such as tumor volume warrants further investigation. Validation in larger, multicenter cohorts will be essential before such multiparametric approaches can inform organ-sparing decision-making.

## Conclusion

In this study, no single ADC strategy was both sufficiently reproducible and discriminative for treatment response assessment in MIBC patients undergoing neoadjuvant immunotherapy.

Whole-tumor measurements were highly reproducible but lacked predictive value, while the most hypointense region showed promising discriminative performance but poor inter-reader agreement. Future research should focus on developing automated, predefined subregional ADC biomarkers that combine the biological sensitivity of targeted measurements with reproducibility required for organ-sparing decision-making, validated across multicenter cohorts.

## Supplementary information

ELECTRONIC SUPPLEMENTARY MATERIAL

## Data Availability

The data that support the findings of this study are available from the corresponding author upon reasonable request.

## Abbreviations

ADC: Apparent diffusion coefficient
AJCC: American Joint Committee on Cancer
AUC: Area under the curve
CI: Confidence interval
DWI: Diffusion-weighted imaging
ICC: Intraclass correlation coefficient
mIF: Multiplex immunofluorescence
MIBC: Muscle-invasive bladder cancer
MRI: Magnetic resonance imaging
nADC: Normalized apparent diffusion coefficient
NAC: Neoadjuvant chemotherapy
pCR: Pathological complete response
RC: Radical cystectomy
ROC: Receiver operating characteristic
ROI: Region of interest
TME: Tumor microenvironment
TURBT: Transurethral resection of bladder tumor
VI-RADS: Vesical imaging-reporting and data system
ypT: Pathological T-stage after neoadjuvant therapy
