# Supplementary material for: Reproducibility and diagnostic performance of ADC for assessing response to neoadjuvant immunotherapy in muscle-invasive bladder cancer: a multi-ROI comparison analysis from the phase-Ib NABUCCO trial
Source: Insights Imaging. 2026 Oct 13;17:255. doi: 10.1186/s13244-026-02397-z (PMC13661850; doi:10.1186/s13244-026-02397-z)
Supplement: Supplementary file 1 [file 13244_2026_2397_MOESM1_ESM.pdf]

Reproducibility and diagnostic performance of ADC for assessing response to neoadjuvant immunotherapy in muscle-invasive bladder cancer: A multi-ROI comparison analysis from the phase-Ib NABUCCO trial

Electronic Supplementary Materials

1. mpMRI protocol

Table S1. mpMRI acquisition protocols for the NABUCCO study.

Patient Preparation & General Setup	
**Bladder preparation**	Bladder emptied + 2 glasses water 30 min before scan
**Antispasmodic**	IV Butylscopolamine bromide 20 mg
**MRI systems**	Philips 3T Achieva dStream / Ingenia
**Coil**	Body coil
**T2-Weighted Imaging**	
**Sequence type**	T2 TSE
**Planes**	Axial, sagittal, coronal
**Slice thickness**	3 mm
**Resolution / voxel size**	0.7 mm
**TR**	5670–9800 ms
**TE**	120 ms
**Diffusion Weighted Imaging (DWI)**	
**Plane**	Axial
**Slice thickness**	3 mm
**Voxel size**	2.3 mm
**TR / TE**	Not specified
**b-values**	0, 200, 800, 1000 s/mm^2
**Dynamic Contrast Enhanced (DCE) MRI**	
**Contrast agent**	Gadoterate meglumine 15 mL (0.5 mmol/mL)
**Injection rate**	3 mL/s + 20 mL saline
**Sequence**	Axial fat-suppressed Dixon
**Temporal resolution**	6 s (20 acquisitions)
**Slice thickness**	2 mm

**Voxel size**	2 mm
**TR / TE**	TR 3.9 ms, TE1 1.38 ms, TE2 2.4 ms

2. Average-rater reliability analysis

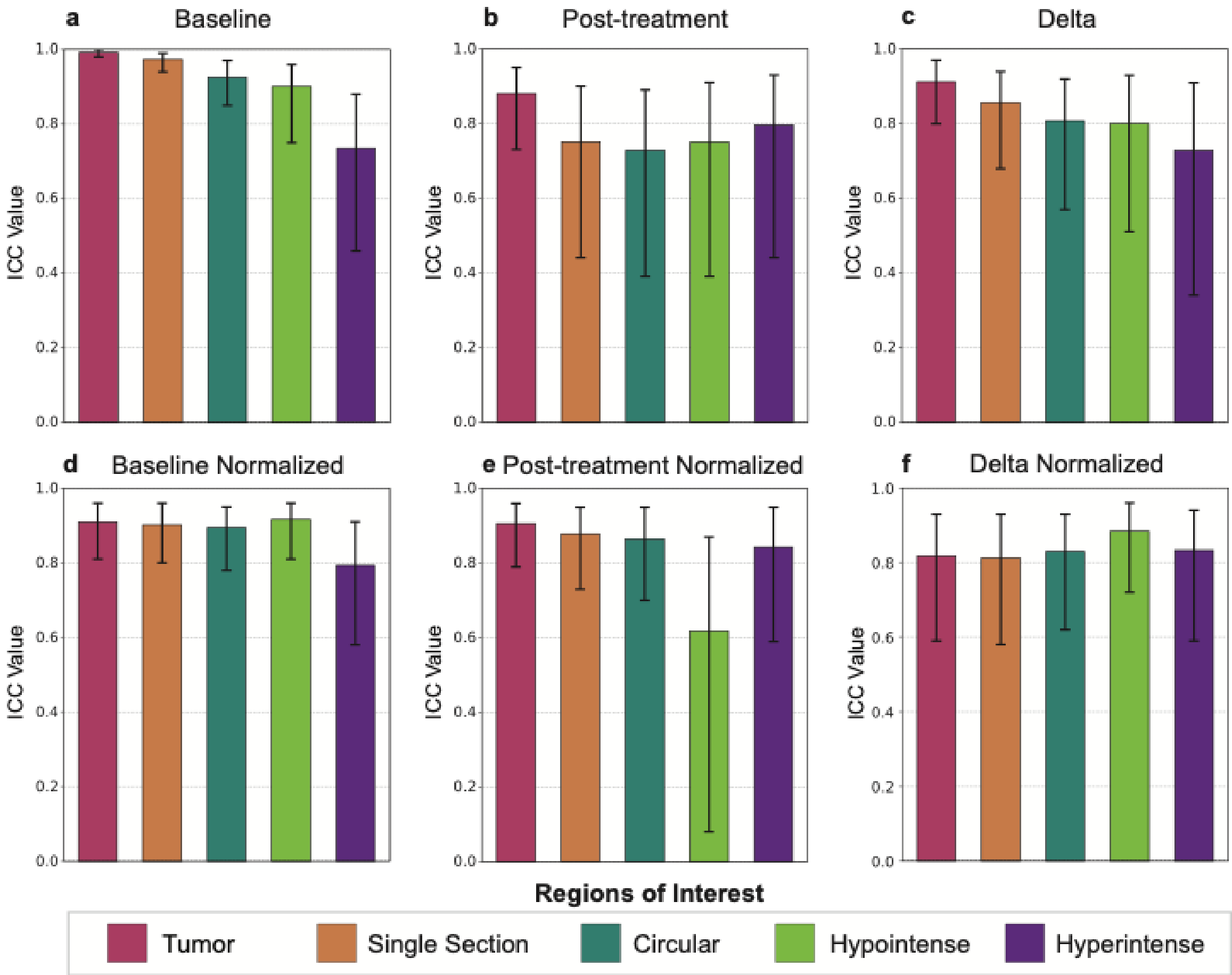

Figure S2_1: Average inter-observer variability of ADC measurements across different ROI strategies and timepoints. Bar plots show intraclass correlation coefficients (ICC[2,k]) with 95% confidence intervals for five ROI methods at (a) baseline (n=25), (b) post-treatment (n=21), and (c) as the percentage change (%ΔADC) (n=21). Panels (d–f) display the corresponding ICCs for normalized ADC values using muscle tissue as reference.

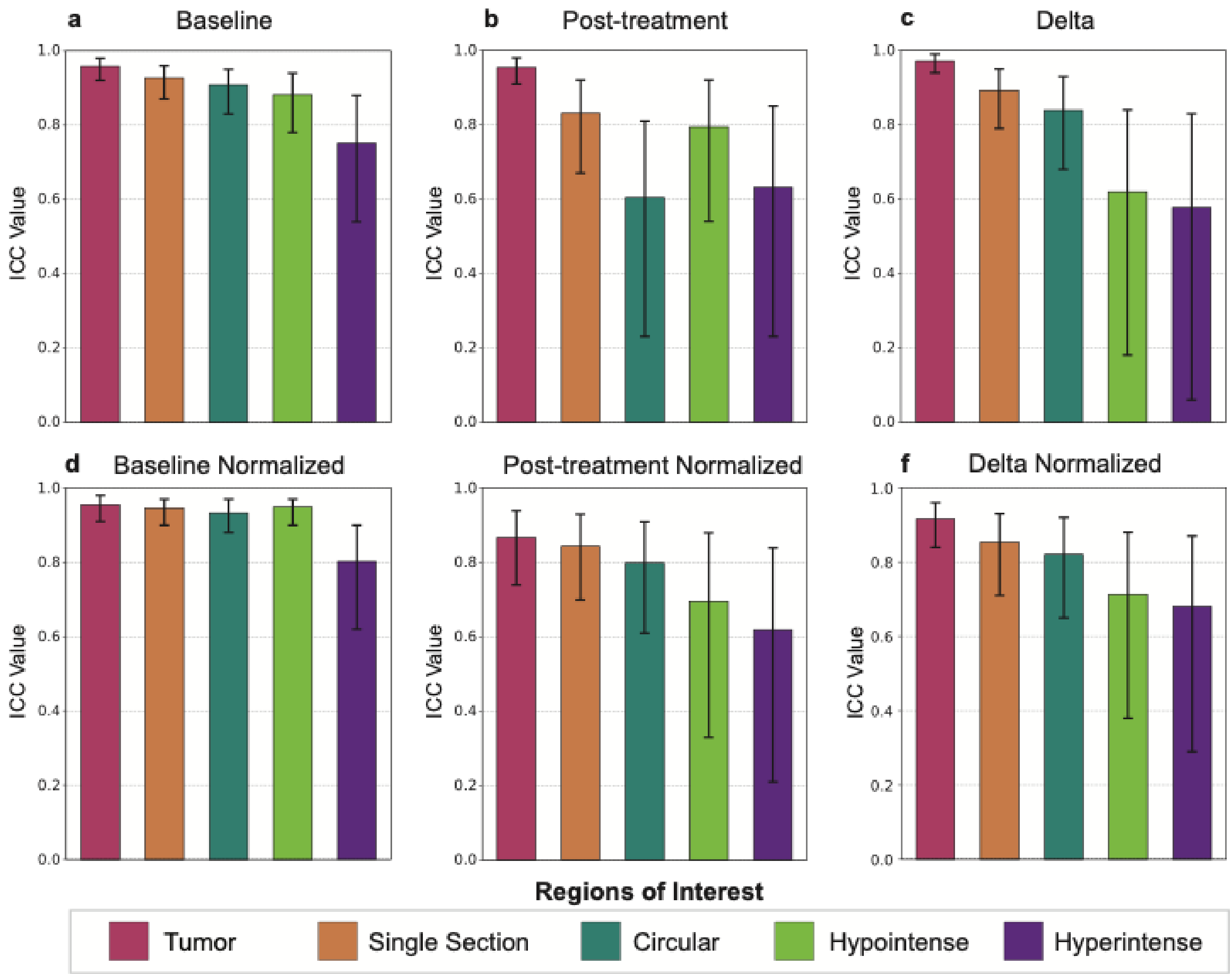

Figure S2_2: Average inter-observer variability of ADC measurements across different ROI strategies and timepoints, for subset analysis. Bar plots show intraclass correlation coefficients (ICC[2,k]) with 95% confidence intervals for five ROI methods at (a) baseline (n=34), (b) post-treatment (n=29), and (c) as the percentage change (%ΔADC) (n=29). Panels (d–f) display the corresponding ICCs for normalized ADC values using muscle tissue as reference.

3. Subanalysis for the ADC maps generated with subset b=0-800 s/mm²

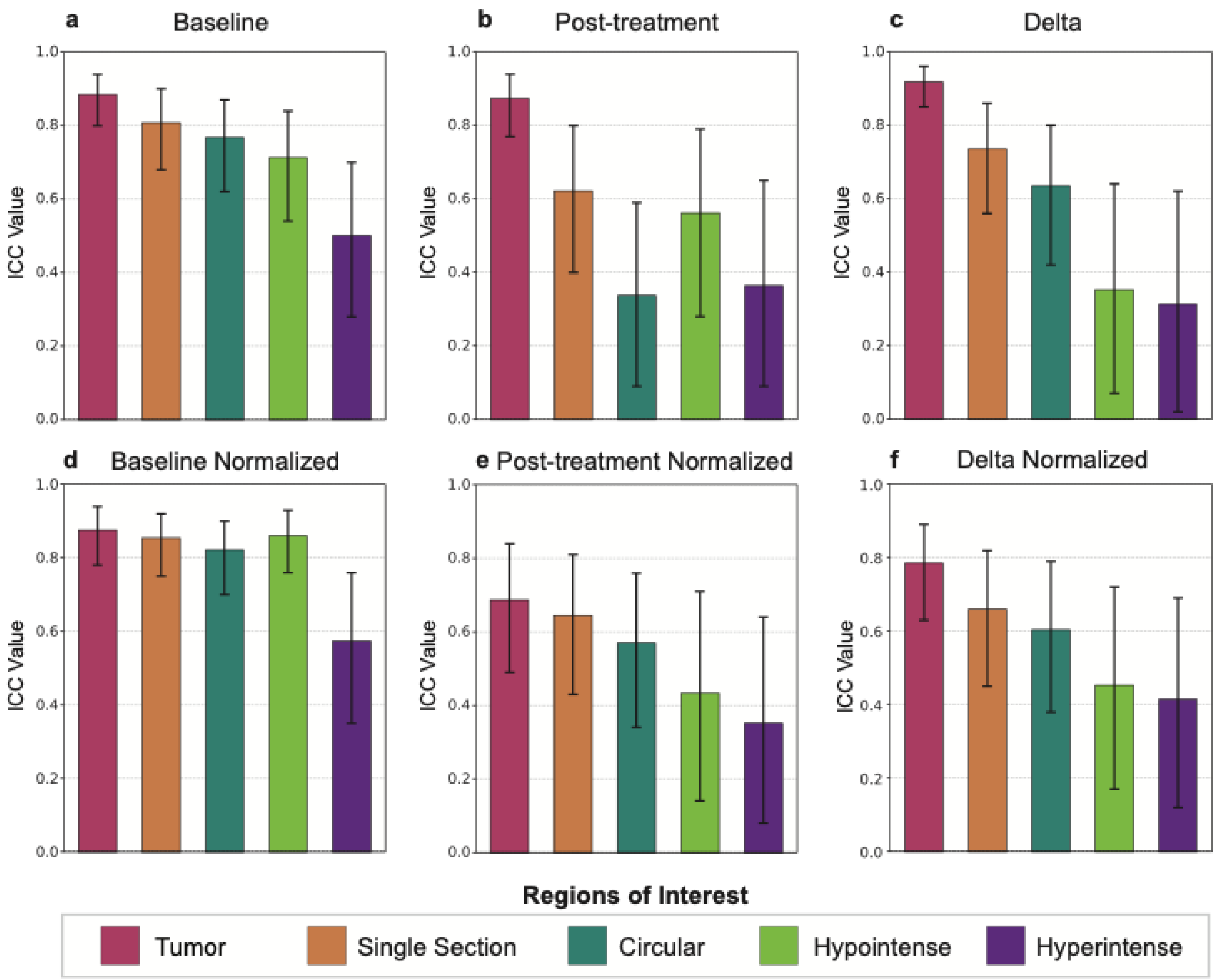

Figure S3_1: Inter-observer variability of ADC measurements across different ROI strategies and timepoints, for subset analysis. Bar plots show intraclass correlation coefficients (ICC[2,1]) with 95% confidence intervals for five ROI methods at (a) baseline (n=34), (b) post-treatment (n=29), and (c) as the percentage change (%ΔADC) (n=29). Panels (d–f) display the corresponding ICCs for normalized ADC values using muscle tissue as reference.

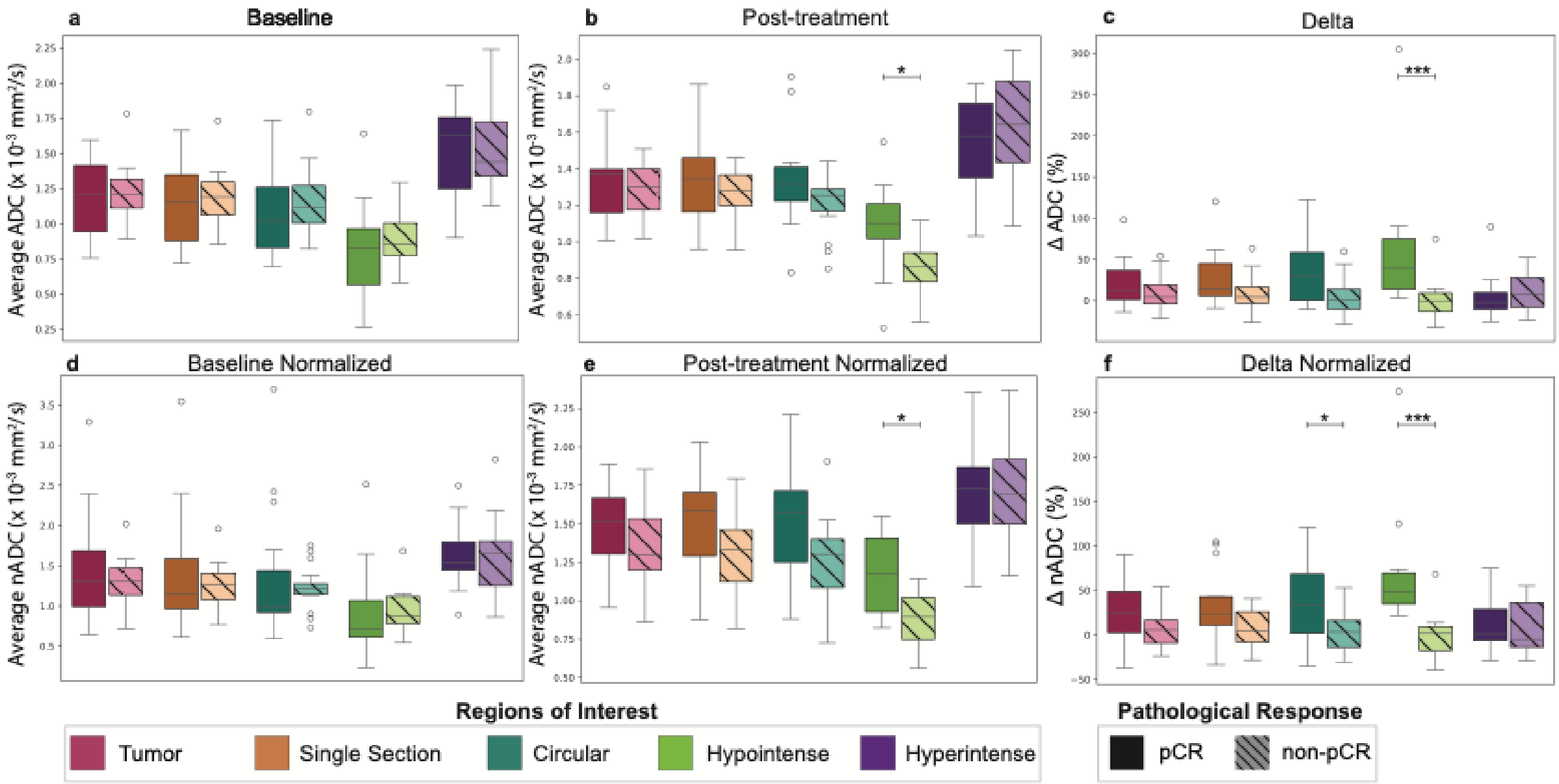

Figure S3_2. Boxplots of ADC values across ROIs, timepoints, and pathological response, for subset analysis. Consensus median ADC values (mean of three readers) are shown for five ROI types at (a) baseline (n=34), (b) post-treatment (n=29), and (c) as the percentage change (%ΔADC) (n=29). Panels (d–f) show the same metrics after normalization to muscle tissue (nADC). Boxplots represent the interquartile range (IQR), horizontal lines indicate the median, and whiskers extend to 1.5xIQR. Pathological complete response (pCR) was defined as ypT0/ypTcis; non-pCR as ≥ypTa. P-values were calculated using the Mann-Whitney U test; * $p < 0.05$; ** $p < 0.01$, *** $p < 0.001$

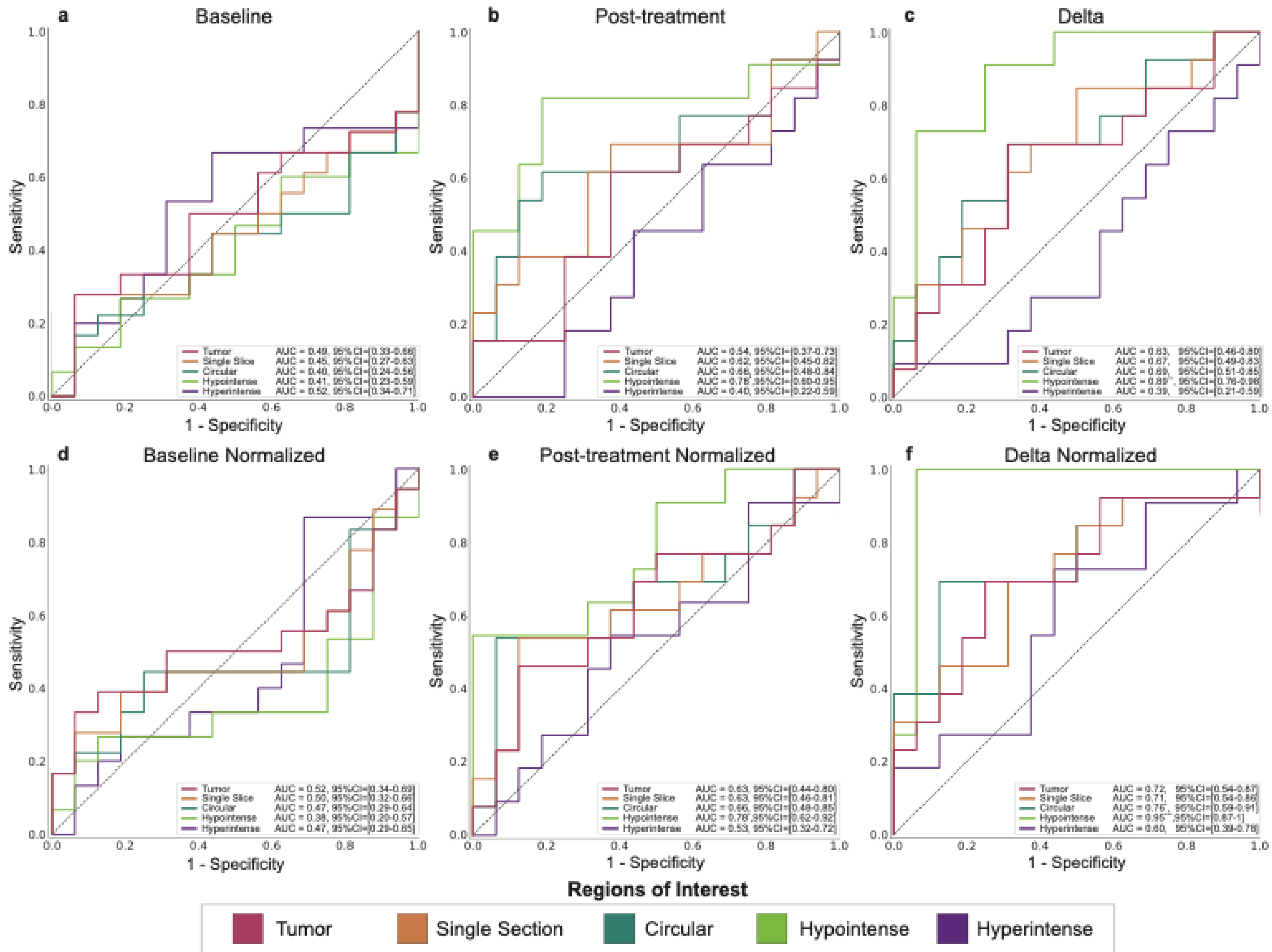

Figure S3_3. Receiver operating characteristic (ROC) curves for the prediction of pathological complete response (pCR) using ADC metrics across ROI strategies and timepoints, for subset analysis. ROC curves with corresponding area under the curve (AUC) and 95% confidence intervals are shown for five ROI types at (a) baseline (n=34), (b) post-treatment (n=29), and (c) percentage change (%ΔADC) (n=29). Panels (d–f) present the same analysis after normalization (nADC). pCR was defined as ypT0/ypTcis; non-pCR as ≥ypTa. Significance test * $p < 0.05$; ** $p < 0.01$, *** $p < 0.001$

4. Correlation between ADC metrics and individual phenotype cell densities from mIF

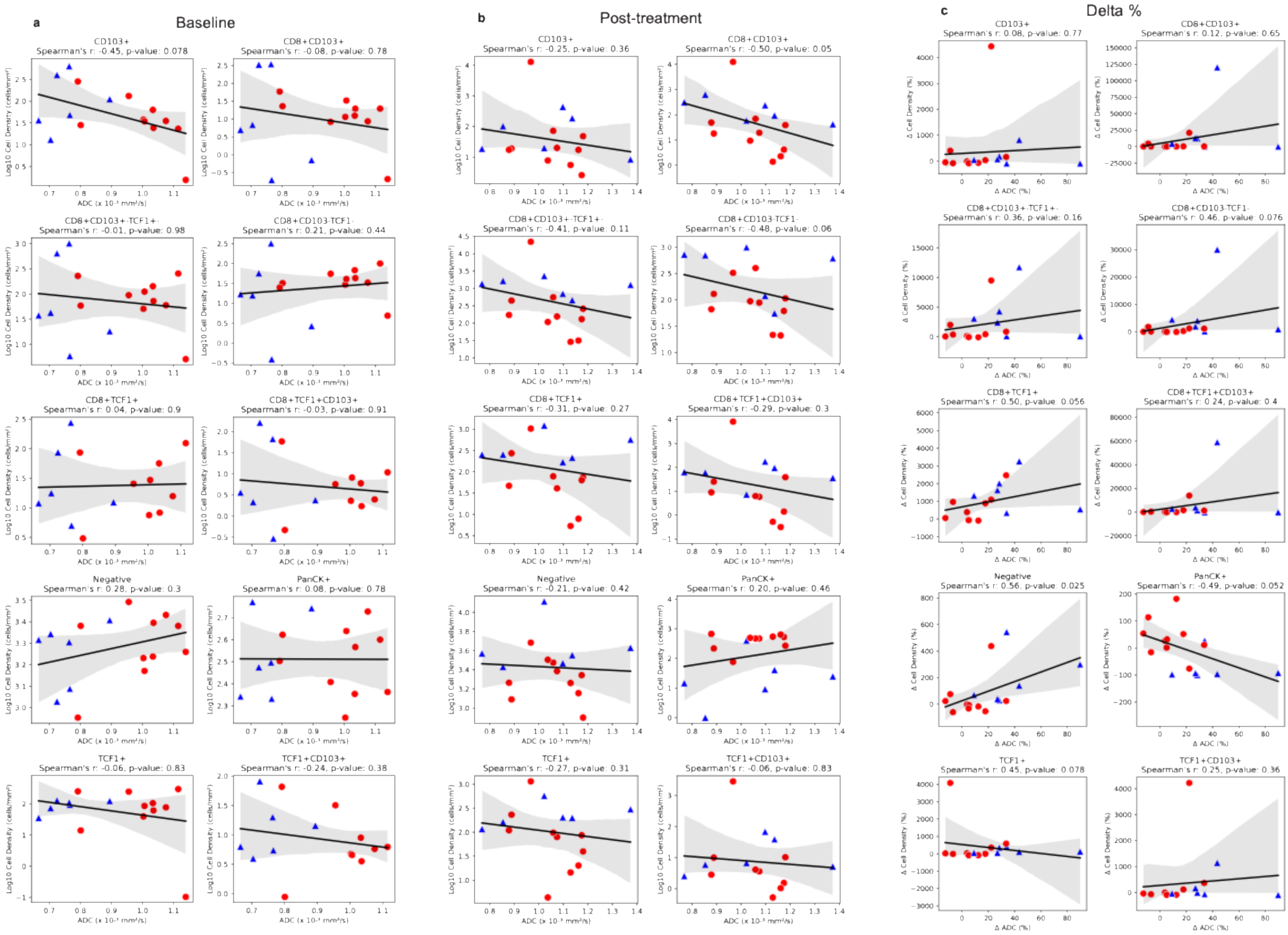

Figure S4_1. Exploratory correlation between whole-tumor ADC metrics and individual phenotype cell densities derived from multiplex immunofluorescence (mIF) (n=20). Scatterplots display the relationship between whole-tumor ADC measurements (given in x10-3 mm^2/s) and per phenotype cell density (cells/mm^2) at (a) baseline, (b) post-treatment, and (c) as percentage change (%ΔADC). Data points are colored by pathological response: pCR (ypT0/ypTcis, blue) and non-pCR (≥ypTa, red). Correlation coefficients (r) and p-values were calculated using Spearman's rank correlation.

Table S2. Median apparent diffusion coefficient (ADC) values for baseline, post-treatment and the percentage change over time (%ΔADC), for subset analysis. For both the raw ADC and the normalized ADC values (nADC). Mean ADC are given in x10^{-3} mm^2/s with 95% Confidence Intervals. P-values refer to comparison between pathological complete response (pCR) and non-pCR, tested with the Mann-Whitney U test. Significant results (p<0.05) are highlighted in **bold**.

* The hypointense and hyperintense have a different patient count for pCR: Baseline n=15, Post-treatment n=11, %ΔADC n=11

Region of Interest	Baseline			Post-treatment			%ΔADC		
	pCR n=18	*non-pCR n=16*	*p-value*	*pCR n=13*	*non-pCR n=16*	*p-value*	*pCR n=13*	*non-pCR n=16*	*p-value*
Median ADC									
**Tumor**	1.21 [0.94-1.41]	1.21 [1.11-1.32]	0.96	1.37 [1.16-1.40]	1.30 [1.20-1.53]	0.71	11.60 [1.57-36.42]	4.44 [-3.98-19.27]	0.23
**Single Section**	1.15 [0.88-1.35]	1.19 [1.06-1.30]	0.62	1.35 [1.16-1.44]	1.28 [1.20-1.37]	0.26	14.32 [5.90-44.99]	4.45 [-3.21-16.68]	0.12
**Circular**	1.02 0.83-1.26]	1.11 [1.00-1.27]	0.34	1.32 [1.22-1.41]	1.25 [1.17-1.29]	0.14	30.45 [0.38-58.48]	1.17 [-9.88-13.81]	0.09
**Hypointense***	0.83 [0.57-0.97]	0.86 [0.77-1.00]	0.40	**1.10 [1.02-1.21]**	**0.86 [0.78-0.94]**	**0.015**	**39.98 [14.14-74.92]**	**-0.55 [-13.19-8.94]**	**0.00087**
**Hyperintense***	1.63 [1.25-1.75]	1.44 1.34-1.73]	0.83	1.58 [1.35-1.76]	1.64 [1.43-1.88]	0.39	-2.61 [-10.47-9.38]	7.03 [-7.79-27.93]	0.36
Median nADC									
**Tumor**	1.30 [0.99-1.69]	1.30 [1.14-1.47]	0.85	1.52 [1.30-1.67]	1.30 [1.20-1.53]	0.23	24.38 [2.67-48.50]	5.60 [-9.14-16.52]	0.051

**Single Section**	1.14 [0.97-1.59]	1.26 [1.08-1.41]	0.99	1.59 [1.29-1.70]	1.33 [1.12-1.46]	0.23	23.71 [10.77-42.92]	4.81 [-7.92-26.15]	0.056
**Circular**	0.99 [0.93-1.44]	1.22 [1.14-1.28]	0.80	1.57 [1.25-1.71]	1.30 [1.09-1.40]	0.14	**33.48 [1.89-68.39]**	**3.48 [-13.63-16.26]**	**0.019**
**Hypointense***	0.71 [0.62-1.07]	0.88 [0.79-1.12]	0.26	**1.18 [0.93-1.41]**	**0.90 [0.75-1.02]**	**0.017**	**48.40 [34.64-69.69]**	**1.77 [-17.80-9.12]**	**0.000087**
**Hyperintense***	1.54 [1.44-1.80]	1.66 [1.26-1.81]	0.77	1.73 [1.50-1.87]	1.69 [1.50-1.92]	0.82	1.24 [-6.35-28.80]	-5.24 [-13.49-36.09]	0.42
